# Doctor retention and distribution in post-apartheid South Africa: tracking medical graduates (2007–2011) from one university

**DOI:** 10.1186/s12960-019-0439-4

**Published:** 2019-12-16

**Authors:** Ann George, Duane Blaauw, Jarred Thompson, Lionel Green-Thompson

**Affiliations:** 1 Centre for Health Science Education, Faculty of Health Sciences, University of the Witwatersrand, 29 Princess of Wales Terrace, Parktown, Johannesburg, South Africa; 2 Centre for Health Policy, School of Public Health, Faculty of Health Sciences, University of the Witwatersrand, 27 St Andrews Road, Parktown, Johannesburg, South Africa; 3 School of Medicine, Sefako Makgatho Health Sciences University, Molotlegi Street Ga-Rankuwa, Pretoria, South Africa

## Abstract

**Background:**

Doctor emigration from low- and middle-income countries represents a financial loss and threatens the equitable delivery of healthcare. In response to government imperatives to produce more health professionals to meet the country’s needs, South African medical schools increased their student intake and changed their selection criteria, but little is known about the impact of these changes. This paper reports on the retention and distribution of doctors who graduated from the University of the Witwatersrand, South Africa (SA), between 2007 and 2011.

**Methods:**

Data on 988 graduates were accessed from university databases. A cross-sectional descriptive email survey was used to gather information about graduates’ demographics, work histories, and current work settings. Frequency and proportion counts and multiple logistic regressions of predictors of working in a rural area were conducted. Open-ended data were analysed using content analysis.

**Results:**

The survey response rate was 51.8%. Foreign nationals were excluded from the analysis because of restrictions on them working in SA. Of 497 South African respondents, 60% had completed their vocational training in underserved areas. At the time of the study, 89% (444) worked as doctors in SA, 6.8% (34) practised medicine outside the country, and 3.8% (19) no longer practised medicine. Eighty percent of the 444 doctors still in SA worked in the public sector. Only 33 respondents (6.6%) worked in rural areas, of which 20 (60.6%) were Black. Almost half (47.7%) of the 497 doctors still in SA were in specialist training appointments.

**Conclusions:**

Most of the graduates were still in the country, with an overwhelmingly urban and public sector bias to their distribution. Most doctors in the public sector were still in specialist training at the time of the study and may move to the private sector or leave the country. Black graduates, who were preferentially selected in this graduate cohort, constituted the majority of the doctors practising in rural areas. The study confirms the importance of selecting students with rural backgrounds to provide doctors for underserved areas. The study provides a baseline for future tracking studies to inform the training of doctors for underserved areas.

## Background

The retention of health professions graduates, especially doctors and nurses, in regions in which they have been trained has become a concern globally, particularly in South Africa (SA) [1]. The migration of doctors to developed countries threatens the delivery of optimal and equitable healthcare in low- and middle-income countries [2–5]. This brain drain represents a huge cost for these countries, reducing their return on investment for the training of doctors [6, 7]. Price and Weiner [8] report that the South African state makes a substantial contribution to the 6-year training of a doctor, which, in 2015, was estimated at R1.3 million [9]. The 2018 Academy of Science of South Africa (ASSAf) Consensus Report on reconceptualising South African health professions education recommends the tracking of graduates in order to influence both the selection and education of medical students and to provide evidence that graduates are impacting on service delivery in rural and underserved areas [10]. Graduate tracking studies provide information on what “graduates do once in practice” [11] and whether medical schools are “training a fit-for-purpose health workforce distributed according to population need” [12].

South Africa has a shortage of doctors [13, 14], with a persistent ratio of less than one doctor per 1000 population between 1996 (0.59) and 2016 (0.8) [15]. This ratio compares favourably with the 2004 sub-Saharan average (0.214) but unfavourably with the Organisation for Economic Co-operation and Development (OECD) average (3.4), ranking SA as having the 16th worst doctor: patient ratio of 67 countries [16]. Recent data suggest an upward trend in this ratio (0.91 in 2017) [17]. The Human Resources for Health (HRH) Strategy for the Health Sector 2012/2013–2016/2017 [18] reported that, between 2002 and 2010, the number of South African medical practitioners increased from 7291 to 11 664, and the number of medical specialists increased from 3585 to 4513. However, there was still a need for an additional 4294 medical practitioners and 7471 medical specialists to meet the country’s needs [18]. SA also has an inequitable distribution of doctors between the public and private sectors [14, 18], with 30% of the doctors working in the public sector [19]. Like other countries, the rural areas in SA are historically under-served [20, 21].

Doctor migration from SA may be a contributing factor to the shortage of doctors [14, 18, 22]. It is difficult to measure the impact of migration and to observe trends since SA does not monitor doctor emigration [23]. The lack of data from SA and other developing countries [6] creates a reliance on figures reported by the destination countries. Such reports suggest that South African doctors make up a large proportion of the workforce in several countries, including the United Kingdom (UK), Australia, Canada, and the United States of America (USA) [4, 6, 24]. There are indications that the migration rates reported in the period following the change in political dispensation from the apartheid regime to democracy in 1994 are declining. In 2000, SA was placed 8th out of the top 10 countries for numbers of emigrating doctors [22], and a 2003 study reported that 30% of doctors had left SA [4]. More recent estimates range between 21 and 29% [25], with more reports of ‘return migrants’ [26], that is people who work outside of the country and then return. One third of the respondents in a 2013 survey had returned to South Africa, with most doctors having spent less than 3 years working in the UK [26]. In a 2016 survey, 37% of 754 South African trained doctors had worked outside SA for periods of up to 5 years.

One of the responses to the doctor shortage has been to increase admissions to the country’s nine medical schools [27]. Despite the increased intake, the goal of increasing the number of medical graduates from 1200 to 1300 per annum to 2400 per annum [28] has not been realised [18]. The figure of 1200 graduates per annum “is viewed as a grossly inadequate production rate for a country with a population of approximately 55 million” [23]. Community service has been proposed as a mechanism to improve the recruitment and retention of healthcare professionals in rural and remote areas [29]. In 1998, a compulsory community service year was introduced for South African medical graduates. The community service year follows a 2-year internship introduced in 2008, prior to which a 1-year internship was required [30]. Indications are that South African doctors tend not to remain in the public sector after completing their community service [31]. The HRH Strategy for the Health Sector 2012/2013–2016/2017 estimated that only about 35 of the 1200 medical graduates per annum remain in rural areas in the longer term [18].

The medical school at the University of the Witwatersrand (Wits University), established in 1919, is responsible for 13% of the annual national first-year intake [27]. In 2003, as part of the global trend towards innovative medical education, Wits University embarked on a hybrid problem-based curriculum from the third year of study [32]. The programme was committed to graduating doctors with core competencies, which included clinical competency and cultural safety, as well as the capacity to work in a plurality of environments and in facilities with variable access to resources [32]. This change was accompanied by selection criteria that were broadened to allow preferential admission to applicants from the previously disadvantaged Black^1^ and Coloured^1^ population groups [27]. The first graduates from this pioneering degree emerged in 2006. The most recent tracking study of medical graduates from Wits University was conducted in 2005 by Price and Weiner [8] using a doctor population based on statutory registration with the Health Professions Council of South Africa and Wits University alumni records from 1960 to 1994. There is no data that addresses the impact of the racially based selection criteria on where these doctors end up working. This paper is the first to report on the outcomes for doctors from Wits University admitted after the changes in curriculum and selection procedures in the early 2000s. The objectives of the study are to describe the cohort of doctors who graduated between 2007 and 2011 in terms of their demographics, the locations of their internship and community service years, and their current work settings (foreign, urban, and rural).

## Methods

This study is a cross-sectional descriptive email survey.

Ethical approval to track and survey medical graduates from Wits University between 2007 and 2011 was granted by the Human Research Ethics Committee (Medical) of the University of the Witwatersrand (Clearance Certificate: M160962). The university registrar granted access to the student database for the 988 graduates from this period. All medical graduates from 2007 to 2011 were eligible for inclusion. A confidentiality agreement was signed between the researchers and the university’s Office of Alumni Affairs to access the alumni register for this graduate cohort.

### Tracking the graduates and non-responders

A combination of telephoning and bulk-messaging was used to verify graduates’ email addresses in the student database and the alumni register. We searched for graduates with missing or incorrect contact details on the Internet and sent messages to those with Facebook and LinkedIn accounts, requesting their email addresses.

Based on information obtained from family members, or locations found via Internet searches and on social media platforms, non-respondents’ locations were recorded as either in or outside the country. These locations allowed an estimate of how many of the non-respondents were still in the country.

### Development and administration of the questionnaire

The online questionnaire was developed and administered using SurveyMonkey®. The survey was piloted with ten recent graduates from Wits University and modified according to the feedback obtained. The survey was conducted at the end of 2016 (5 years after the last graduating cohort). The recruiting email included an information sheet and an electronic link to the survey, with a statement that completion of the questionnaire indicated consent.

The questionnaire consisted of three sections with 39 open- and closed-ended questions extending over four pages: demographics (7 questions), work history and current working conditions (21 questions), student experiences (10 questions), and an optional request to provide a name or student number. All survey items had to be answered, except for the optional one. Closed-ended questions included a non-response option and allowed only one response. Respondents were able to review their answers prior to submission. Participants reported on the location of their internship and community service and, if they were still practicing medicine, about their current workplaces (country, sector, and area). This paper reports on data from the 28 questions in the first two sections of the questionnaire.

### Statistical analysis

Unique respondents were identified using the Internet Protocol (IP) address assigned in SurveyMonkey®. The most recent response was retained for one duplicate response. Tracking, response, and completion rates were calculated for the final dataset. Relevant data from the first two sections of the survey (demographics and work history) were analysed using Stata 14 (StataCorp., USA). Frequency and proportion counts and multiple logistic regressions of predictors of working in a rural area were conducted. Open-ended data were grouped into categories using inductive content analysis [33].

## Results

Of the 988 medical graduates from 2007 to 2011, one has since died. We were unable to trace 199 graduates due to incomplete or incorrect information in the student database and alumni register. The email invitation was sent to the remaining 788 graduates (79.8%), who had been tracked.

**Fig. 1:**
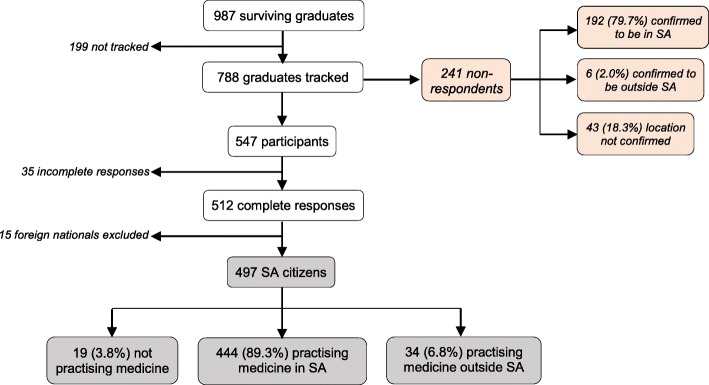
Tracking and survey results for 987 surviving graduates (2007–2011)

The response rate for the survey was 69.4% (547/788) (see Fig.1), with 35 incomplete surveys. Overall, 51.8% (512/987) of the total graduate group completed the survey. Fifteen foreign nationals were excluded from the analysis because of restrictions on them working in South Africa [34]. Responses from the remaining 497 South African citizens (hereafter referred to as the ‘respondents’) were analysed (see Fig. 1).

There were 241 (30.6%) non-respondents to the survey (see Fig. 1). However, information from family members, the Internet, and social media searches allowed us to determine that 79.7% (192/241) of the non-respondents were still living in South Africa, with 2% (6/241) outside the country. We could not confirm the locations of 18.3% (43/241) of the non-respondents (see Fig. 1).

We located 710 graduates, of whom 89.6% (636/710) were in South Africa. Even if one assumes that the 277 with unknown locations (sum of 199 not tracked, 43 with unconfirmed locations and 35 incomplete responses) have all left the country, at least 64.4% of the total cohort were still in the country at the time of the survey.

Table 1 shows the number of respondents for the years 2007–2011. The mean response rate per graduating year was 50.2% (range 43.1–61.1). The 497 respondents were representative of the graduate cohort in terms of gender (predominantly female) and background (predominantly urban background) (see Table 2), based on self-reported data in the questionnaire.

**Table 1:** Respondents by graduating year (2007–2011)

Year	Graduate cohort	Respondents	Percentage
2007	174	75	43.1
2008	189	100	52.9
2009	194	93	47.9
2010	223	102	46.2
2011	208	127	61.1
Total	988	497

**Table 2:** Demographics for the 497 respondents

Variable	Number of respondents	Percentage of respondents	Percentage of graduate cohort
Gender			
Female	305	61.4	60.5
Male	192	38.6	39.3
Population group			
Black	127	25.6	32.3
Coloured	12	2.4	2.3
Indian	116	23.3	26.2
White	237	47.6	39.0
Other	5	1.0	0.2
Background			
Urban	411	82.7	80.9
Rural	86	17.3	11.4
Unknown			7.8

### Internship and community service locations

Four of the 497 respondents did not complete an internship: two completed their internship abroad and two no longer practise medicine. Table 3 shows the level of the healthcare facility for the internship and community service period for the 493 respondents who completed their internship. Level 1 or district hospitals offer limited specialist medical services compared to level 2 (regional) and level 3 (tertiary) hospitals [35]. Fourteen respondents did not complete the mandatory community service (the four who had not completed their internship and ten doctors who emigrated after completing their internship). Fewer than 25% of the 493 respondents completed an internship at level 1 and 2 hospitals, while 62% completed their community service year at facilities at these levels (see Table 3).

**Table 3:** Internship and community service locations for 493 respondents

Level of hospital	Internship	%	Community service	%
Level 1 (district hospital)	16	3.2	177	36.8
Level 2 (regional hospital)	105	21.5	123	25.4
Level 3 (tertiary/ central hospital)	370	75.1	156	32.2
Specialised hospital	1	0.2	2	0.4
Other	0	0	25	5.2

### Current work settings

#### Respondents not practising medicine

Nineteen of the 497 South African citizens (3.8%) were not practising medicine at the time of the survey (see Fig. 1). Three respondents were temporarily not practising because they were either on maternity leave or awaiting employment. Respondents who had left the profession reported working in the pharmaceutical and healthcare industries, and for non-governmental organisations, with two specialising in public health.

#### Respondents practising medicine outside South Africa

Thirty-four (7.1%) of the 478 respondents still practising medicine work in countries other than South Africa (see Table 4), with 76.5% of these doctors in Canada, the UK, Australia, or the USA.

**Table 4:** Locations of doctors working outside South Africa (*n* = 34)

Country	*n*	%
Canada	8	23.5
UK	7	20.6
Australia	7	20.6
USA	4	11.8
Germany	2	5.9
Israel	2	5.9
Denmark	1	2.9
Namibia	1	2.9
Other	2	5.9

**Fig. 2:**
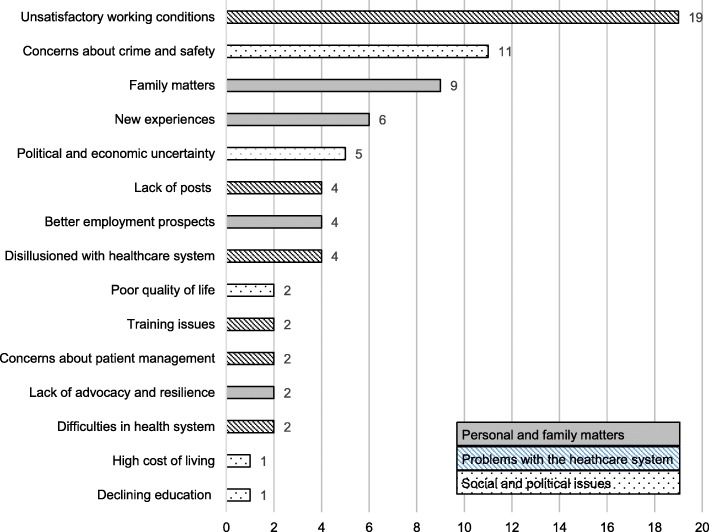
Reasons for leaving South Africa

The reasons (*n* = 74) respondents gave for leaving South Africa were categorised into three broad categories, as shown in Fig. 2: *problems with the healthcare system* (*n* = 33), *personal and family matters* (*n* = 21), and s*ocial and political issues* (*n* = 20). *Unsatisfactory working conditions* (*n* = 19) was the most frequently cited reason for leaving, followed by *concerns about crime and safety* (*n* = 11) and *family matters* (*n* = 9).

Ten of the 34 respondents working outside South Africa planned to return, one after specialising abroad, three to study further, and the remainder for various reasons, including “to live in Cape Town”. The reasons offered by the 17 respondents not intending to return included having settled in their chosen country, family matters, better career opportunities and working conditions in the host country, and the social and political conditions in South Africa. Seven respondents living outside the country were undecided about their plans.

#### Respondents practising medicine in South Africa

Of 415^2^ doctors working in South Africa, 28% (116) were generalists (self-employed, employed, or medical officers^3^), while 59.8% (248) were intending to specialise (53) or in the process of specialising (195). Most worked in the public sector (79.5%; 330).

Ninety-two percent (384) of 417^4^ respondents worked in urban areas and 33 (6.6%) in rural areas (self-reported data). Comparing the characteristics of the urban and rural doctors shows that rural doctors are disproportionately male, Black, and of rural origin (see Table 5).

**Table 5:** Characteristics of urban and rural workers (*n* = 417)

Variable	Urban	Rural	*p* value^^^
Gender			
Female	247 (64.3%)	15 (45.5%)	0.039
Male	137 (35.8%)	18 (54.6%)	
Population group*			
Black	94 (24.5%)	20 (60.6%)	< 0.002
Coloured	11 (2.9%)	0 (0%)	
Indian	95 (24.5%)	4 (12.1%)	
White	182 (47.5%)	9 (27.3%)	
Graduating year			
2007	60 (15.7%)	1 (3.0%)	0.126
2008	78 (20.4%)	6 (18.2%)	
2009	69 (18.0%)	4 (12.1%)	
2010	81 (20.9%)	9 (27.3%)	
2011	96 (25.1%)	13 (39.4%)	
Background			
Urban	330 (89%)	12 (36.4%)	< 0.000 1
Rural	54 (14.1%)	21 (63.6%)	

^^^Fisher’s exact test

*Two respondents reported their population group as ‘other’

The multiple logistic regression analysis of predictors for working in a rural area of South Africa at the time of the study indicates that exposure to a rural area increases the chances of doctors returning to these areas (see Table 6). A rural background, an internship in a rural hospital, and completing their community service in a rural hospital all, independently, increased the probability of currently working in a rural area. A rural background was the strongest predictor of working in a rural area: respondents from a rural background were nearly five times more likely to be working in a rural area. An internship or completing community service in a rural facility increased three-fold the likelihood of returning to work in a rural area. The number of years since graduation and the doctors’ gender were not significant predictors of working in a rural area. Race was excluded from the multiple regression analysis because of the multi-collinearity between race and rural origin.

**Table 6:** Predictors of currently working in a rural area in South Africa—multiple logistic regression

Variable	OR	95% CI	*p* value
Years since graduating	0.76	[0.558; 1.047]	0.094
Male	1.248	[0.542; 2.874]	0.603
Rural background	4.884	[1.982; 12.031]	0.001
Internship in rural area	3.119	[1.232; 7.900]	0.016
Community service in rural area	3.057	[1.164; 8.029]	0.023

## Discussion

This is the first tracking study of medical graduates from Wits University in more than 20 years. Just over half (51.8%) of the graduates from 2007 to 2011 participated in this survey. A best-case scenario, taking into account the graduates that could not be traced, and the non-responders outside South Africa, is that 64.4% of the graduates (455 respondents and 192 non-respondents; 647) were practising medicine in the country at the time of the study. Those in South Africa worked in generalist and specialist environments (28.0% and 10.6% respectively), with almost half (47.7%) in trainee specialist appointments or intending to specialise (13.0%). These trends are similar to those reported by Price and Weiner [8] in their 2005 study, using data from 1960 to 1994. Their study demonstrated that graduates spent a large part of their early careers in the public sector. Price and Weiner found that 63% of the most recent graduates in their population (1990–1993) were still in the public sector. A vast majority of these graduates worked in the larger metropolitan areas, and only 14% worked in rural areas or small towns. This study highlights an increase in the proportion of female graduates, from 10% in the 1960s and 45% in the 1990s to more than 60% in both the 2007–2011 respondent and graduate cohorts. It further highlights that more than half of the respondents (60.7%) were trainee specialists or intending to specialise. Doctors who are training as specialists are more likely to work in urban and private settings because of higher salaries [8], contributing to ongoing shortages in the public service and rural areas. In the Price and Weiner study, which had follow-up times longer than this study, 40% of the medical graduates from Wits University between 1960 and 1994 had specialised. Notwithstanding the possibility of different definitions of ‘rural’ used in the two studies, only 6.6% of the respondents in this study worked in rural areas compared to the 14% reported as working in rural areas or small towns in the Price and Weiner study.

Only 34 (6.8%) of the 497 respondents have left the country. The potential migration rate of 35.6% for the total graduate cohort, assuming that all respondents not tracked and the non-respondents are outside of the country, is higher than recent estimates of migration rates of about 30% [14, 18], but lower than the 46% reported for the period 1960–1993 [36]. The cross-sectional nature of this study may not reflect the final destinations of participants. Many respondents are still specialising and may yet leave the country, while 10 out of the 34 doctors abroad (29%) indicated their intention to return to SA. The international destinations, whether temporary or final, for medical graduates from Wits University remain largely unchanged, although the USA, previously reported as the most popular destination [36], has been surpassed by Canada, the UK, and Australia.

Doctors’ reasons for leaving their home countries are a complex mix of push and pull factors [37–40]. Previous studies suggest that South African doctors are more likely to leave because of push factors [37, 41]. The 29 doctors in the 2005 study by Bezuidenhout et al. listed financial reasons, working conditions, and the rate of crime and violence as their reasons for leaving the country [41]. Crime and safety issues were also a major push factor reported in a study of 653 South African doctors living in Australia [37]. The main reasons for relocating cited in this study, namely, unsatisfactory working conditions, concerns about crime and safety, and family matters, are similar to those previously reported. The formidable problems of how to resolve the political and workplace issues identified as push factors are often beyond the control of educational institutions [36] but promoting advocacy and resilience in undergraduates is a potential area to target in medical education programmes [42]. The 2015 Australian study by Greenhill et al. [43] identified three components of resilience in medical students in longitudinal integrated clerkships: a student support network, sustained educator support and guidance, and promoting student development of strategies to cope with adversity. These three components of resilience suggest possible strategies that could be incorporated into undergraduate programmes, and faculty professional development and support programmes.

The redistribution of respondents to underserved areas from internship to community service mirrors the findings of a longitudinal study reviewing the first 15 years of community service nationally, which found that more doctors were being allocated to rural areas and district hospitals [30]. However, the question is whether these doctors are being retained post-community service. A 2014 report suggested that community service professionals were not being retained in the public sector, with about 20% indicating that poor working conditions could influence them to leave the country [18]. In our study, rural vocational placements and a rural background increased the likelihood of working in a rural area, confirming what has been shown in other studies [44, 45]. In this study, a rural background was found to be a highly significant predictor (OR 4.998) for working in a rural area. Wits University introduced rurality as a selection criterion in 2015, indicating a need for graduate tracking to assess where these future graduates will end up working. The disproportionate representation of Black graduates among those who work in rural areas may justify the broadening of the selection criteria to give preference to the selection of Black graduates in this cohort. A planned follow-up of this cohort will confirm whether this trend is sustained beyond the early period following graduation.

Health workforce planning is crucial to health system planning [23]. Reid [31] pointed out that the community service policy was initiated “in the absence of a broader human resources for health (HRH) strategy for the health sector”. The current HRH Strategy 2012/2013–2016/2017 came into effect “after the community service programme had already been institutionalised” [31]. The new HRH strategy, currently in preparation, needs to provide a policy framework to improve community service, which could contribute to greater retention of doctors in rural areas.

This study is limited firstly, by a possible selection bias in favour of the graduates who remained in South Africa and were, therefore, easier to locate. The bias is mitigated by having data on the locations of non-respondents and using that data to estimate the number of doctors likely to be in the country, assuming that all those who could not be located had left South Africa. Secondly, a limited analysis was possible because of the small numbers of graduates from rural backgrounds in this graduate cohort. The number of graduates from rural backgrounds is expected to increase in future cohorts because of the recent introduction of rurality as a selection criterion, which should allow future analyses to confirm the findings of this study.

## Conclusions

This cross-sectional study provides information about a 5-year cohort of medical graduates from the University of the Witwatersrand. These graduates were among the first to graduate from an innovative curriculum that aimed to broaden the range of graduate competencies to meet the country’s needs. The results show larger numbers of doctors from this university remaining in South Africa than in previous tracking studies. The majority of these doctors were in specialist training at the time of the study, which did not explore their intention to leave after their training period. The study confirms the importance of selecting students with rural backgrounds to promote the number of doctors working in rural areas. Black graduates, who were preferentially selected in this graduate cohort, are disproportionately represented among the doctors practising in rural areas at the time of the survey.

In line with the ASSAf Consensus Report calling for more graduate tracking studies to influence both the selection and education of medical students and to provide evidence that graduates are impacting on service delivery in rural and underserved areas, this study highlights some strategies that could be implemented. There is a need to implement strategies to foster greater resilience in undergraduate students and for better health workforce planning to maximise the retention of medical graduates in underserved areas. Workforce planning needs to be informed by more graduate tracking studies, especially longitudinally, from Wits University and other South African medical schools, to observe the impact of changes in national and institutional policies on doctor retention and distribution and to inform selection procedures to improve health care delivery in underserved areas. This study has established a large cohort of graduates that can be followed up for tracking the career trajectories of its graduates emerging from a transformed curriculum.

## Data Availability

The dataset supporting the conclusions of this article is available in the Open Science Framework repository, https://osf.io/g5r6n/?view_only=cbd7374a0db14dcb89be50b52e500d8c

## Abbreviations

ASSAf: Academy of Science of South Africa
CI: Confidence interval
IP: Internet Protocol
SA: South Africa
UK: United Kingdom
USA: United States of America
Wits University: University of the Witwatersrand

## Footnotes

1 The racial classifications of Black and Coloured (mixed race) were introduced during the apartheid era (1947–1994) according to the Population Registration Act (No. 30 of 1950). The terms are still in use to assist the South African government with redress of previously disadvantaged population groups.

2 Discordant figures are due to responses that could not be used.

3 The term ‘medical officers’ is used to refer to generalists who work in the public sector [23].

4 Discordant figures are due to responses that could not be used.
